# Women’s worries about prenatal screening tests suspected of fetal anomalies: a qualitative study

**DOI:** 10.1186/s12905-023-02211-8

**Published:** 2023-02-13

**Authors:** Seyyedeh Mahboubeh Mirtabar, Zeynab Pahlavan, Sajedeh Aligoltabar, Shahnaz Barat, Fatemeh Nasiri-Amiri, Maryam Nikpour, Fereshteh Behmanesh, Sina Taheri, Khadijeh Nasri, Mahbobeh Faramarzi

**Affiliations:** 1 Research Clinical Psychology, Student Research Committee, Health Research Institute, Babol University of Medical Sciences, Babol, Islamic Republic of Iran; 2 Rouhani Hospital, Babol University of Medical Sciences, Babol, Islamic Republic of Iran; 3 Student Research Committee, Babol University of Medical Sciences, Babol, Islamic Republic of Iran; 4 Infertility and Health Reproductive Research Center, Health Research Institue, Babol University of Medical Sciences, Babol, Islamic Republic of Iran; 5 Social Determinants of Health Research Center, Health Research Institue, Department of General Courses, Babol University of Medical Sciences, Babol, Islamic Republic of Iran; 6 Non-Communicable pediatric Disease Research Center, Health Research Institute, Babol University of Medical Science, Babol, Islamic Republic of Iran; 7 Department of Gynecology and Obstetrics, Arak University of Medical Sciences, Arak, Islamic Republic of Iran

**Keywords:** Worry, Pregnant women, Screening tests, Fetal anomalies, Coping strategies

## Abstract

**Background:**

Pregnant women with suspected fetal anomalies experience a great deal of stress following prenatal screening tests. The present study aimed to investigate women's worries about prenatal screening tests suspected of fetal anomalies.

**Methods:**

Through the use of qualitative content analysis, the reports of women whose prenatal screening tests were suspected of fetal anomalies were analyzed and the results were interpreted. The participants were selected from four public and private maternity care clinics of Babol, Iran, from December 2021 to January 2022, using targeted convenience sampling. Data were collected from 20 women aged 24 to 41 years old, who underwent prenatal screening tests and were suspected of fetal anomalies, using semi-structured face-to-face interviews.

**Results:**

The four main themes included the "causes of worries" (with sub-themes of challenge with spouse and relatives, medical diagnosis processes, previous unpleasant experiences, physical and mental problems, financial worries, and misinformation), "anxiety-coping styles" (with cognitive emotion-oriented, behavioral emotion-oriented, and problem-oriented sub-themes), "reactions to a possible diagnosis of anomaly" (with three sub-themes, namely concealment, extreme fear and worry, and denial), and "attribution of the cause of the anomaly" (with sub-themes of consanguine marriage, evil eyes, tendency to have a baby of a particular gender, a history of anomalies in the previous child, the spouse's medical illness, unplanned pregnancy, and high maternal anxiety).

**Conclusion:**

Women with suspected fetal anomalies experience a great deal of stress, the most important reason for which is the "physician’s uncertainty". "Sharing worries with relatives" was the most common style of coping with worries. Establishing emotional support and empathetic communication between midwives and physicians with pregnant women suspected of fetal anomalies were important ways to reduce their worries.

**Supplementary Information:**

The online version contains supplementary material available at 10.1186/s12905-023-02211-8.

## Background

Congenital anomalies are believed to be the leading cause of perinatal mortality. Based on WHO, one-fifth to two-fifths of infant mortality is attributed to congenital anomalies [1]. Over the recent decades, prenatal screening for the diagnosis of fetal anomalies has been offered as an option for all pregnant women during prenatal care visits [2]. In Iran, approximately 98% of women undergo ultrasound screening at 18 weeks of gestation [3]. In approximately 5% of pregnancies, routine fetal ultrasound screening finds an abnormal fetal appearance. Suspected fetal anomalies require further diagnostic measures. Subsequent steps need to be taken to determine whether this diagnosis is a false-positive screening, or a chromosomal anomaly and true positive screening [4]. Studies have indicated that these diagnostic follow-ups may have adverse psychological consequences for pregnant women [5].

Despite significant advances in prenatal diagnosis tests, little attention has been paid to the psychological consequences of these tests. Women’s potential concern about having a diagnostic test and waiting for a result is often overlooked. A report indicated that several women experienced a negative psychological reaction after receiving the result of a suspected anomaly test [6]. Women with suspected fetal anomalies may have numerous psychological problems, one of which is that they mostly have optimistic expectations of routine ultrasound screening and are thus rarely prepared for adverse findings [7]. It has been reported that some women do not undergo additional fetal screening tests due to worries about definitive results [8]. There is also the issue of ongoing uncertainty between the suspected diagnostic test and the final diagnosis [9]. Additionally, women's worries increase if the diagnosis of anomalies is definitive. There are two dire consequences for mothers undergoing definitive diagnoses of fetal anomalies: losing a fetus with congenital anomalies by having a selective abortion and accepting a child with special needs.

Evidence has shown that women with suspected fetal anomalies experience the highest level of worry twice, once immediately after the diagnosis of anomalies by the physician, and once again, during the termination of pregnancy [10]. A study emphasized that hospitalized women experienced strong feelings of sadness and grief for termination of pregnancy with abnormal fetuses [11].

Pregnant women with suspected fetal anomalies will have numerous problems with the postpartum period after a long period of great worry if they give birth to abnormal fetuses. There is evidence also that maternal stress levels may remain high during and after pregnancy due to the diagnosis of fetal anomalies [12]. Coppola et al. [13] found that parents of sick children experience higher levels of anxiety, depression, and stress compared to those of healthy ones. A study found that mothers of children with congenital heart diseases were significantly more likely to have postpartum anxiety than those of healthy children. Furthermore, the severity of fetal anomalies was associated with the severity of maternal depression [14]. A study by Nes et al. (2014), on postpartum women in Norway (17 to 36 weeks after delivery), assessed postpartum psychological distress with congenital anomalies. They indicated that the rate of maternal psychological distress was high due to the birth of a child with severe anomalies, such as Down syndrome. The birth of an abnormal infant with a treatable disease, such as clubfoot, had a temporary and insignificant effect on maternal depression [12].

Some studies have addressed the concerns of women diagnosed with fetal abnormalities [15]. But there are little information about the type of concerns of women who are suspected of being pregnant with an abnormal fetus. This study has been directed toward the causes of the worries and the relevant coping strategies among women with suspected fetal anomalies. The present study aimed to detect worries of pregnant women with suspected fetal anomalies and the way of coping with them.

## Methods

### Design

The present qualitative study included a conventional content analysis. We followed the guidelines of the Consolidated criteria for reporting qualitative research (COREQ) for reporting of study [16]. We put the checklist in the Additional file 1: Appendix.

### Settings and participants

The study was conducted on 20 eligible pregnant women, below 20 weeks of pregnancy, singleton pregnancy, and suspected to have abnormal fetuses in four perinatology centers in Babol, including two public hospitals (Rohani and Yahyanejad), and two private clinics, from December 2021 to January 2022. The inclusion criteria were the age of above 18 years old, being diagnosed as suspected to fetal anomaly, speaking Persian fluently, and consenting to participation.

Herein, fetal anomalies were diagnosed by gynecologists and perinatologists based on the criteria of diagnostic screening tests in the first or second trimester of pregnancy with moderate or high risk. In the prenatal diagnostic centers in Iran, the first-trimester screening is performed between the 11th and 13th week of pregnancy; it includes Biochemical check marker (Beta HCG free and PAPP-A) and nuchal translucency (NT). Based on the results of the first-trimester screening tests, a pregnant woman is classified as one of the high-risk, medium-risk, or low-risk groups in terms of having an abnormal fetus. If the first-trimester screening result is low-risk, there is no need for the second-trimester screening. If a person is classified as high-risk, a diagnostic test, such as chorionic villus sampling (CVS) or amniocentesis, is immediately recommended in order to make a definitive diagnosis. If the screening result suggests a borderline risk, the physician usually recommends a second-trimester screening (16 to 20), including the blood test of four markers (inhibin a, estriol, Beta HCG, and AFP), ultrasound scan anomaly, amniocentesis, and cell-free DNA, depending on the problem [17].

### Data collection

The interviews were conducted by a clinical psychologist (first author) with pregnancy-related expertise, who was trained by an expert psychologist of the research team (Author, MF) before the study. The purposeful sampling was used until data saturation. At four perinatology centers, the interviewer explained the goals and the research procedure to 50 eligible pregnant women who had screening tests with suspected fetal anomalies, 25 of whom refused to enter the study. Their reasons for not entering the study were: not having time for a one-hour conversation with a psychologist, not being satisfied with recording the interview, and feeling anxious talking about their concerns.

The researcher conducted clinical interviews with individuals who consented to participate, and the codes were saturated for up to 20 patients. Some of the interviewer's questions from the patients are as follows:

- What was your reaction when you found out that your fetus might be abnormal?
- How did you feel after the result of the suspected fetal abnormality screening test?
- What concerns did you have when you found out that your fetus might be abnormal?
- What was your husband's reaction to the results of the screening tests?
- What did you expect from your doctor regarding this problem?
- How have you coped with the problem of possibly having an abnormal fetus?
- Furthermore, the entire interview session was recorded so that the relevant themes could be reviewed by the research team. The in-depth interviews began with semi-structured questions. The interviews lasted for an average of 30 to 45 min and were recorded after obtaining the written consent from the participants. Data collection was performed until data saturation (3 consecutive interviews with no new themes).

### Trustworthiness of the data

In a qualitative study, four criteria (reliability, confirmability, transferability, and responsiveness) are used to assess reliability or coherence [18]. The following strategies were used to ensure the reliability of this study. First, according to the research question, conventional content analysis methods were utilized under the careful supervision of the research team. Secondly, all the interviews were conducted by a trained interviewer, a clinical psychologist, to perform qualitative research and interviewing techniques. Thirdly, the interviewer had a long-term interaction with the data. The researcher built trust and connection with the participants, considered a variety of perspectives, and jointly built meaning from the concerns. The first 10 interviews were independently coded by six authors, after which the interpretations were compared. If the interpretations differ, they are discussed until reaching a consensus. At the end of the study, membership verification and peer verification methods were used. In the member verification method, a printed copy is returned to the participants to match the accuracy of the data with their experience. In the peer-review method, transcripts, codes, and categories were sent to five impartial professors of obstetrics, psychology, and fertility to review the reliability of the categories, following which sub-categories are extracted. Fourth, the researcher attempted to select the participants with diversity of age, place of residence, income level. Finally, in addition to audio recordings and transcripts, multiple data sources, including field notes, observations, memos, and journals, were used.

### Data analysis

The obtained data were analyzed using the conventional content analysis with MAXQDA software (version 18, VERBI software, Berlin, Germany). After each interview, the answers were typed word by word in an MS Word document (2018 version) and entered into MAXQDA software (version 18). Subsequently, the transcribed text was divided into semantic units and the concepts reflected in the text were summarized. The semantic units were shortened and named using appropriate codes. The codes were divided into sub-sets based on similarities and differences, and then the categories were extracted. Ultimately, the themes were extracted based on the concepts identified in the text.

## Results

Table 1 presents the demographic characteristics of the studied women. Following data analysis, 51 codes, 12 sub-themes, and four themes were obtained. Table 2 depicts the four themes, including the causes of worries, coping strategies, reactions towards a possible diagnosis of anomaly, and attribution of the cause of the anomaly.

**Table 1:** Demographic characteristics of the population study

Participant number	Age	Educational level	Occupational status	Gravid	Gestational age (weeks)
1	41	Diploma	Self-employed	4	14
2	27	Diploma	Employee	2	20
3	32	Diploma	Self-employed	2	17
4	24	BS	Self-employed	2	19
5	35	BS	Working	3	11
6	24	Diploma	Self-employed	1	17
7	25	BS	Self-employed	2	15
8	32	BS	Working	1	16
9	32	BS	Self-employed	3	14
10	32	BS	Employee	2	20
11	28	Diploma	Self-employed	1	28
12	30	Diploma	Self-employed	2	14
13	38	BS	Self-employed	2	15
14	39	BS	Employee	2	14
15	35	Diploma	Employee	6	19
16	35	Diploma	Self-employed	4	11
17	24	BS	Self-employed	1	18
18	34	BS	Self-employed	4	14
19	25	BS	Working	2	14
20	30	Diploma	Self-employed	1	13

**Table 2:** Themes, sub-themes, codes, and frequencies extracted from the data analysis

Themes	Sub-themes	Final codes
Causes of worry	Challenge with spouse and relatives	Negligence
		Not spending enough time
		Not taking the possibility of anomaly tests seriously
		Not allowing an abortion if the anomaly is proven
		Misinformation about anomalies
		Lack of husband’s family understanding Current status
	Medical diagnostic processes	Delay in test results
		The physician’s uncertainty
		Uncertainty in the physician's statements
		The physician’s improper clarification
	Previous unpleasant experiences	Loss of the previous child
		Consequences of abnormal infant parents among the acquaintances
		Existence of family problems
		History of high-risk pregnancies
		History of abortion or abnormality in the previous child
	Physical and mental problems	Previous medical problems of the woman
		Difficulty in decision-making about abortion as the fetus ages
		Physical and psychological changes in women during pregnancy
		Older age
	Financial concerns	High costs of additional tests
		High cost of amniocentesis
		Costs for multiple visits
	Inappropriate information	Insufficient information about anomalies
		Maternal misinformation about abnormalities
		Getting information about anomalies from cyberspace and the Internet
Coping styles with worry	Cognitive emotion-orientation	Reading books
		Listening to motivational voices
		Gratitude and appreciation
		Praying
		Trust in God
		Trust in the physician's current diagnosis
		Sharing the problem of fetal anomalies with relatives
	Behavioral emotion-orientation	Participation in childbirth preparation classes
		Doing physical activities and sports
	Problem orientation	Referral to another physician for a more detailed examination
Reactions towards the possible diagnosis of anomalies	Hiding the problems	Not sharing problems with friends or relatives
		not sharing problems with the spouse
	Fear and worry	Severe fear after diagnosis of an anomaly
		Severe worry after diagnosis of an anomaly
		Worry about taking good care of a previous healthy child
		Fear of the consequences of having an abnormal infant
		Uncertainty and confusion in decision making
	Denial	Performing additional tests
		Probability of mistakes in test answers
Attributing the cause of the anomaly	Personal beliefs	Consanguine marriage
		Devil eyes of others
		Tendency to have a baby of a certain gender (boy or girl)
		A history of abnormalities in the previous child
		Spouse's medical illness during pregnancy
		Unplanned pregnancy

### Causes of women’s worries about suspected fetal anomalies

This was the most frequent theme among the four main themes. The six sub-groups of causes for worries included challenges with the spouse and relatives, medical diagnosis processes, previous unpleasant experiences, physical and mental problems, financial worries, and misinformation. The medical cases sub-theme was the most common cause of worries, and the "physician unreliability" was the most common code released.

### *Challenges with the spouse and relatives

A major cause of worry in these women was found to be the challenge with their husbands and relatives after being informed about the possibility of having an abnormal infant. A number of them stated that relatives did not understand their critical status. Furthermore, their husbands did not have enough time and support for them. Husbands had little knowledge about the test process and the possibility of abortion, and sometimes refused legal permission for abortion after the finalization of the tests and abortion orders.

I did not talk to anyone about the test of my infant's anomalies because my previous child had a complete defect in the auricle and was born at 36 weeks. I was very annoyed by my relatives because when the child was asleep, they would make a sound with plastic to see if the child can hear. I was very upset about what they did, and now I get nervous when those memories come to my mind. Relatives do not help anything, but exacerbate the situation. (p4).

My husband is very busy and cannot take me to the doctor. I try not to be bothered too much because my annoyance causes the fetus to be bothered. The husband's financial status is very important. Many families may have insufficient money and may be bothered. All these imposes a lot of pressure on the pregnant mother. People's living conditions are different. Some women go to the doctor with their husbands, but my husband is busy. (p11).

### *Medical diagnosis processes

Delay in the preparation of test results, physician’s uncertainty, ambiguity in the physician's statements, and inappropriate behavior of the physician in expressing the problems were found to be the other causes of these women's worries. The majority of the participants believed that the physician’s certainty and resolving medical ambiguities would be conducive to reducing their anxiety. On the contrary, inappropriate behavior of the physician and delay in the preparation of test results would increase their worry.

I did the screening tests in two centers. The first laboratory did not explain the test results at all, but the second laboratory explained everything very well. When individuals' information is more accurate, they are less stressed. (p9).

The doctor's talk before the amniocentesis helped me a lot to make the amniocentesis easier. The doctor's relationship with the patient is very important. (p6).

### *Previous unpleasant experiences

Loss of a previous child, the consequences of being the parents of an abnormal baby among the acquaintances, family-related problems, a history of high-risk pregnancies, and a history of abortion or abnormalities in the previous child cause unpleasant experiences and increase the women’s worries about suspected fetal anomalies.

I'm stressed and worried because I've had several miscarriages. I'm afraid that the baby would have a problem. (p18).

My first baby was healthy. The second time I got pregnant, the doctor said the fetal heart was not yet created and that I had to undergo an abortion. In the third pregnancy, the fetal heart was created and everything was going well. I was shocked and cried when they said that my fetus might have anomalies. (p3).

I was very annoyed with the abortion. I visited several doctors. They do not easily allow abortions. It has to be approved by several specialists. We spent a few days to receive their agreement with the abortion. (p5).

### *Physical and mental problems

Other cases of worries in women with suspected fetal anomalies included having previous physical problems, changes in pregnancy, and old age. As the gestational age increased, the decision to have an abortion seemed to become more challenging for such women.

Thank God as my previous children are healthy, but my age may have caused abnormalities in this child. (p18).

If I had no food craving or absolute rest, and had a normal pregnancy, I would not have any worries, but now, I am deprived of being active because of my status. It makes me sick. Everyone eats and I cannot eat well. I'm not healthy. I do not like this status. I want to be healthy and do my son's work. I used to do all the work myself, but now I can't. My son has become nervous. (p13).

### *Financial worries

The high cost of perinatal diagnostic tests, including amniocentesis, as well as the cost of multiple doctor visits was the concerns of women with suspected fetal anomalies.

We just built a house. The cost of tests was too high. I could not do the tests completely. (p17).

My husband is a worker. When I was told to do the screening tests again, I could not pay for it; so, my husband told me to visit the doctor and ask which test was more important to do, and then do it." (p14).

The costs are very high. The costs of commuting, tests, and doctor visit are high, and they become staggering when we should pay all this together. (p10).

### *Misinformation

Insufficient and on a number of occasions, wrong information about anomalies, and getting information from cyberspace, the Internet, and uninformed people are some issues that increase the worries of pregnant women with suspected fetal anomalies.

I wanted to have ultrasonography again, maybe it had a problem. My relative was told to have an abnormal baby, but the baby was healthy when born. I searched about amniocentesis, and I know it is related to Down syndrome. (p6).

I do not know what amniocentesis is. I'm going to do a Google search. I've only heard its name once. I'm very worried as they said the risk of fetal anomalies is high. (p13).

The relatives' misinformation is very important. It stresses a person out because it is affected by old beliefs. Relatives have no knowledge and cannot behave properly and help the stress of a pregnant mother. (p11).

### Coping styles in women with suspected fetal anomalies

This theme included three sub-themes, namely cognitive emotion-oriented, behavioral emotion-oriented, and problem-oriented sub-themes. The emotion-oriented coping style was the most common released code. The most frequent codes in this type of coping included sharing the problem of fetal anomalies with relatives, trust in the doctor's diagnosis, and trust in God.

### *Emotional-cognitive coping strategies

Coping strategies in this group of women included reading books, listening to motivational voices, gratitude and appreciation, praying, trusting in God, trusting in the diagnosis of the current doctor, and sharing the problem of fetal anomalies with others.

There is nothing I can do in this situation. I always trust in God in difficulties. Do you think I should have an abortion? (p1).

I think it is better to share the problem of fetal anomalies with the family. Families cheer us up. They accompany and encourage us. I would like the days to pass faster and my mother to come back from the trip so that I could talk to her about this issue to become calm. I pray every night and listen to motivational voices. (p13).

The acquaintances encourage us. How can we carry a big burden (fetal anomalies) alone? Do you think it's possible? Pregnancy itself has its own stress and a pregnant woman is mentally ill. Without the help of my husband and acquaintances, I cannot be saved from the negative feelings caused by this problem (baby anomaly). (p9).

### *Emotional-behavior coping strategies

This type of coping strategy was the least common among these women. Their helped their stress by engaging in certain activities, like attending childbirth preparation classes, physical activity, and exercise.

Doing pregnancy exercises and attending childbirth preparation classes are very effective in improving the pregnancy both mentally and physically. They also have a great effect on the baby's consciousness. The previous baby was very alert at birth unlike some babies who are tired and do not feel well. I think I will feel better if I exercise during this pregnancy. (p9).

### *Problem-solving coping strategies

Some women used problem-solving methods to control their anxiety; for example, a method was to visit another doctor for a more accurate examination.

I researched and found out that the doctor was a perinatologist and I came to her for a more detailed examination. (p8).

As I had problems in the pregnancy of my previous baby, they said they were taking a Chorionic Villus Sampling (CVS) test to make sure my fetus was OK. I also asked my acquaintances and they referred me to this doctor. (p19).

### Women's reaction towards the possible diagnosis of anomaly

Women with suspected fetal anomalies, after being diagnosed, experienced three types of reactions, namely concealment, extreme fear and anxiety, and denial. "Extreme fear and anxiety" was the most common reaction among the three sub-themes.

Some women even hid the presence of an abnormal fetus from their husbands for a long time. Therefore, they alone experienced extreme fear and anxiety about the consequences of having an abnormal baby. Uncertainty and confusion were other reactions in most interviews.

I did not talk about my baby's anomalies to my husband's relatives because they may have a different look at my baby when the baby is born. I prefer my husband's relatives not to know. (p18).

The physician can give us some reassurance; for example, 100 people have done this test, how many of them have received a complete and correct answer? I also asked my doctor if my fetus will be healthy 100%. The doctor said that I must do the amniocentesis, which has more complications. I said it is ok, I accepted the risk, but I want a guarantee. I want to make sure that my baby is healthy. I want my baby to be healthy. I want to make sure that my baby will not suffer for a lifetime. This stress is 100% with us until the end of pregnancy. Every night and day I think that if my baby has a problem, what do I want to do? I had severe stress, and insomnia and was extremely upset. Every moment passed with stress until the test result came. They said it would take three weeks for the answer. I kept calling the laboratory, but it took a month until I was given the initial answer. It took a month and a half for me to get both answers. (p10).

I would like the anomalies test results to be good under any circumstances, but now I say no if under any circumstances, the test results show the slightest problem, and my child has a problem, I can have an abortion as I do not want to have a sick and disabled child for the rest of my life. I know how hard the care of a sick child is. Now, I am worried if the physician does not diagnose the abnormality and I have an abnormal child. The child will suffer later. It is true that my heart will be broken. Abortion will be upsetting, but it is better not to have a baby with disorders. (p3).

Certain women denied the diagnosis of anomalies and sought additional tests or doubted test results. It was hard and painful for them to accept the reality.

I wanted to do an ultrasound again. Maybe the ultrasound was wrong. One of our relatives was told that her baby had a problem, but the baby was healthy when born. I was very hopeful that my re-test will be negative and without any problem. I hope the answer to the first test would be wrong. (p6).

I did ultrasound and laboratory tests. The risk of the interstitial anomaly was reported. The same issue had happened to my relatives, and they underwent amniocentesis, but their baby was healthy. It gave me hope. (p7).

### Attributing the cause of anomalies

Women with suspected fetal anomalies attributed the causes of their anomalies to certain factors, such as consanguine marriage, devil eye of their people around, a desire to have a baby with a particular gender (boy or girl), a history of anomalies in the previous child, a history of the husband’s illness, unplanned pregnancy, and high maternal anxiety. The most common attribution was "a history of anomalies in the previous child" among these women.

We had a consanguine marriage. My first child was also at a high risk of fetal anomalies. The screening test of my second child was “Down syndrome” positive. My first child was healthy. I had the cell-free test for the child, but I had an amniocentesis in this pregnancy, and thank God, It was negative. In general, consanguine marriage is risky. (p10).

Despite the problem of fetal anomalies, it is very important to be understood by our people around. Many of our acquaintances have infertility problems. One of them told me that my baby was a girl, and that having a daughter is very good. “Lucky you!" I hated their behavior. I say to myself again God! you considered me worthy of having children, it has nothing to do with anyone, but there are evil eyes of other people. (p3).

My fetal anomaly problem was due to my husband's varicocele. Do you think that is not possible? (p1)

## Discussion

The present study aimed to investigate women's worries about prenatal screening tests for suspected fetal anomalies. The results revealed that these women experienced great worries while being diagnosed for fetal anomalies. Moreover, different coping styles and various reactions were highlighted.

According to the results of our study, "medical diagnostic processes" were the most common cause of worries in women with suspected fetal anomalies, and the " physician’s uncertainty " was the most common factor increasing women's worries. Other factors, such as obtaining information through personal search, sometimes incorrectly, along with delay in the preparation of diagnostic test results played roles in this issue. Consistent with these results, a qualitative study on 24 pregnant women with a positive diagnosis of fetal anomalies reported that women did not receive the necessary emotional support from their physicians to decide whether to continue or terminate the pregnancy. This study suggested that physicians further empathize with women with diagnosed fetal anomalies and give them medical advice [19]. Other qualitative studies have reported that patients made great efforts to obtain a correct medical diagnosis [20, 21]. Certain papers however contradicted such findings; in a qualitative study on 25 pregnant women diagnosed with fetal anomalies, Irani et al. (2019) reported that mothers were supported by various sources, but mostly by their families. The family's attention and support gave hope and confidence to the mothers; they did not feel alone facing problems of anomalies. Among family members, women considered their husbands to be the important supporters [22]. There are several possibilities concerning the cause of medical concerns of our study patients. Primarily, there is a limited number of specialized centers in our city; in the city of sampling, Babol, there are only two specialized centers for procedures such as amniocentesis and CVS. Therefore, patients may have to wait a few days for preformatting these procedures. Furthermore, the results of the tests are not ready immediately. For these reasons, patients may endure a great deal of stress until the response. Secondly, after receiving the results of the test, patients may be able to visit their specialist and deal with their concerns with a delay of a few hours. Thirdly, some of the women in our study stated that they feel ashamed or stigmatized for carrying an abnormal fetus; thus, they may not be able to raise their concerns even with their spouse and get sympathy or support.

We found that "financial concerns" were the major concerns of pregnant women with suspected fetal anomalies. The high cost of specialized tests was an additional burden on top of the problem of anomalies. Unfortunately, in our country, the insurance systems do not cover specialized abnormality tests and screenings. Thus, the patient has to pay the high costs of tests without any financial support. If special facilities were available for this group of pregnant women who experience abnormal fetuses, less stress would be imposed on them. A US study in 2017 investigated the relationship between financial pressure and birth weight. The participants included 138 pregnant women who completed questionnaires for assessing financial stress, depressive symptoms, pregnancy-specific anxiety, perceived stress, and general anxiety during pregnancy. Their results indicated that financial pressure was positively associated with depressive symptoms, anxiety, perceived stress, and pregnancy-specific distress while being negatively associated with birth weight. Depression mediated the relationship between financial stress and birth weight [23]. The findings suggested that governments and healthcare systems of countries should provide adequate insurance support for women with suspected fetal anomalies in line with the national population policies.

The results of this study demonstrated that women with fetal anomalies practiced coping strategies, including cognitive emotion-oriented strategies (such as praying and trusting in God), behavioral emotion-oriented strategies (participating in childbirth preparation classes, and doing physical activity and exercise), and problem-oriented strategies (visiting another physician for a detailed examination) in order to reduce their worries. Consistent with these findings, a study aimed to determine the coping strategies in pregnant women diagnosed with fetal anomalies, and reported that they visited different physicians, underwent various ultrasound and diagnostic tests, searched for peer experiences, prayed, accepted the fate, relied on the faith, and gained social support to cope with their worries [22]. Religious tendencies and spiritual coping strategies are effective ways to cope with problems because of their important impact on people's lives. Therefore, the quality of life and coping strategies can be ameliorated by improving an individual's religious attitudes [24]. Supporting the present findings, we can refer to Allport's view (2010). He believed that religion and spirituality were comprehensive issues with organized and internal principles, and religious people sincerely believed in their religious teachings. He also held that religion was the only thing that could improve individuals' mental health [25]. Strict abortion laws in Iran intensify the worries of pregnant women with suspected fetal anomalies. In the Islamic country of Iran, abortion is prohibited and considered as a crime. Abortion for maternal reasons is allowed when the mother's life is seriously at risk. Abortion due to fetal abnormalities is allowed under very difficult conditions: when the fetus is under 4 months old and has a severe organ defect.

Our findings indicated that "fear and anxiety" were the most common reactions of pregnant women toward positive or suspected fetal anomaly tests. In this regard, Ekelin et al. (2008) reported that most parents were not prepared for the diagnosis of fetal anomalies and faced high levels of psychological distress. When responding to the crisis, they realized that their sense of security and trust in their physicians was false. Since different caregivers were involved in diagnosing their fetal anomalies, the women needed to receive a single treatment plan and careful caregiver support [26]. Therefore, it seems as if women with suspected fetal anomalies needed psychological support to accept the reality and cope with the crisis of a positive diagnosis of fetal anomalies. Parents need to receive accurate, correct, and timely information from their physicians. Interruption in the continuity of care or incorrect referral centers burdens mothers with additional stress. Accordingly, smooth transfer and clear communication between referral centers is important [27].

Herein, we reported that many participants were worried about their relatives' misjudgment about the fetal anomaly and its cause, thereby hiding their problems. Kamranpour (2020) also indicated that the stigma of abortion and having an abnormal fetus scared women of being judged by relatives. Thus, they could not share grief with them and get adequate support [28]. Support from family and friends are important. Some women of our study were more active in seeking support than others due to their personality traits. It appears that needs vary from one couple to another, between partners, and from one point in time during pregnancy. The following aspects should hence be considered in a structured follow-up program during pregnancy after prenatal diagnosis of the anomaly; written information, access to a secure website with high quality information in your mother tongue, support for parents with similar experiences, and ongoing contact with a specialist liaison nurse with pediatric experience.

According to the results of the present study, some attributions of the cause of anomaly caused a feeling of guilt about fetal anomalies and remorse in women. Consistent with our study, the subjects in the work by Maguire et al. (2015), who underwent elective abortion due to fetal anomalies, blamed themselves for the decision to terminate the pregnancy; consequently, social isolation and memory-related grief was prevalent among them [29]. Another paper, which interviewed parents after diagnosing fetal anomalies, showed that the shock of diagnosis could decrease through the following factors: empathetic, non-judgmental, and professional support, and timely access to further tests and appointments with healthcare providers [30]. In addition to providing counseling services, assessing women's mental health status, holding psychotherapy sessions, considering the effects of such a loss on women and their families, as well as focusing on the upcoming problems facing these women increase their understanding of their new situations and significantly helps them to adapt to their existing status. These women feel guilty about having an induced abortion for their own pregnancy. The results of other research also confirm this finding that receiving emotional support from family and close friends is effective in restoring mental peace and reducing women's anxiety and stress. In fact, these women believed that family support is effective in creating empathy and reducing guilt and self-blame. According to them, in this situation, the cooperation of family members and close friends can help them balance the situation and turn back to normal [31].

The current research did not take into consideration the consequences and concerns after abortion or pregnancy termination due to anomalies. Since several studies have indicated that the related outcomes would persist for more than 1 year [31, 32], limiting the sample to gestational age was a limitation of the present study. Another limitation herein was not investigating the co-occurrence of anxiety in women and husbands with suspected fetal anomalies. Further research could be suggested to investigate the couples' worries about suspected fetal anomalies.

The present study can pave the way for psychiatrists, gynecologists, and midwives toward helping women with suspected fetal anomalies. It is also possible to detect strategies for coping with hidden worries by identifying different aspects of their problems. The research results can help pregnant women with suspected fetal anomalies to identify and apply effective coping strategies in order to prevent the adverse physical and psychological consequences of long-term uncertainty anxiety (suspicious test to birth). Furthermore, certain strategies were suggested herein in terms of coping with worries and improving the physical and mental health of pregnant women with suspected fetal anomalies.

## Conclusions

The obtained findings could be of several clinical applications for midwives, obstetricians, ultrasound specialists, and all maternal care personnel responsible for pregnant women with suspected fetal anomalies. Maternal caregivers need to focus on the important "emotional support" issue as a vital aspect of communicating with these patients. The physicians and midwives, who are responsible towards these women, are suggested to receive adequate training and learn how to inform patients of suspicious or positive test results. It is also necessary for these physicians and midwives to promote empathetic behavior and talk to these patients in order to contribute to reducing their worries.

The present study can help design interventions for providing the optimal care for these women at different times and promoting their mental health by an overview of women's psychological experiences with prenatal screening tests for suspected fetal anomalies. It could be also recommended that midwives and physicians, who provide maternal care for women with suspected fetal anomalies, be aware of the adverse consequences of worries about the birth of a fetus with an anomaly or fetal loss. Further studies are needed to examine efficient strategies for establishing emotional support for these women and reducing their concerns.

## Supplementary Information

**Additional file 1:** Consolidated criteria for reporting qualitative studies (COREQ).

## Data Availability

The datasets used or analyzed in the current study are available from the corresponding author upon reasonable request, but the interviews and all coding steps are in Persian and require translation.
