# Supplementary material for: Women’s worries about prenatal screening tests suspected of fetal anomalies: a qualitative study
Source: BMC Womens Health. 2023 Feb 13;23:66. doi: 10.1186/s12905-023-02211-8 (PMC9926685; doi:10.1186/s12905-023-02211-8)
Supplement: Supplementary file 1 — Additional file 1: Consolidated criteria for reporting qualitative studies (COREQ). [file 12905_2023_2211_MOESM1_ESM.docx]

**Manuscript: Women's Worries about Prenatal Screening Tests Suspected of Fetal Anomalies: A Qualitative Study**

**Consolidated criteria for reporting qualitative studies (COREQ): 32-item checklist**

Developed from:

Tong A, Sainsbury P, Craig J. Consolidated criteria for reporting qualitative research (COREQ): a 32-item checklist for interviews and focus groups. *International Journal for Quality in Health Care*. 2007. Volume 19, Number 6: pp. 349 – 357

| **No. Item** | **Guide questions/description** |
| --- | --- |
| **Domain 1: Research team and reﬂexivity** |  |
| *Personal Characteristics* |  |
| 1. Interviewer/facilitator | One research associate (MM) conducted interviews. She was trained by FM  and received ongoing feedback based on review of transcripts.  Methods, p. 5 |
| 2. Credentials | MM- PhD  PZ-MD  AS-MSc Students  BS-MD  NF-PhD  NM-PhD  BF-PhD  TH-MD  NK-MD  FM-PhD |
| 3. Occupation | MM- PhD candidate for By Research Clinical Psychology  PZ- Obstetrics and Gynecology Specialist, Perinatologist  AS-MSc Student in Counseling Midwifery,  BS- Associate professor of University, Obstetrics and Gynecology Specialist.  NF- Associate professor of University  NM-Researches  BF- Assistant Professor of University,  TH- Physician, health services researcher  NK- Assistant Professor of University, Obstetrics and Gynecology Specialist, Perinatologist  FM- Professor of University |
| 4. Gender | 9 member of the study team identifies as Female and 1 as male. |
| 5. Experience and training | MM- was trained as a clinical psychologist with training in health psychology and with training in qualitative methods.  The research assistants were trained and supervised by MF. |
| *Relationship with participants* |  |
| 6. Relationship established | The interviewer had no prior relationship with the participants. |
| 7. Participant knowledge of the interviewer | As part of providing informed consent, participants received an information sheet that described the goals for the study. In brief, this stated that the purpose of the study was to women's worries about prenatal screening Tests.  Methods, p. 5 ,6 |
| 8. Interviewer characteristics | The interviewer was a trained research assistant who has conducted several studies with the author in the field of mental health of pregnant women. |

| **Domain 2: study design** |  |
| --- | --- |
| *Theoretical framework* |  |
| 9. Methodological orientation and Theory | Framework Method (content analysis)  Data analysis, p. 6 |
| *Participant selection* |  |
| 10. Sampling | The convenience sampling method was used herein and researcher conducted clinical interviews with individuals who consented to participate.  Data collection, p. 5 |
| 11. Method of approach | The researcher conducted clinical interviews with individuals who consented to participate, and the codes were saturated for up to 20 patients. Furthermore, the entire interview session was recorded so that the relevant themes could be reviewed by the research team. The in-depth interviews began with semi-structured questions. The interviews lasted for an average of 30 to 45 minutes and were recorded after obtaining the written consent from the participants. Data collection was performed until data saturation.  Data collection, p. 5,6 |
| 12. Sample size | 20 participants  Data collection, p. 5,6 |
| 13. Non-participation | 25 of eligible patients refused to enter the study. Their reasons for not entering the study were: not having time for a one-hour conversation with a psychologist, not being satisfied with recording the interview, and feeling anxious talking about their concerns.  Data collection, p. 5,6 |
| *Setting* |  |
| 14. Setting of data collection | The interviews were conducted face-to-face in a private room in a dedicated space in 4 perinatology centers.  Data collection, p. 5 |
| 15. Presence of non-participants | No |
| 16. Description of sample | Table 1 presents the demographic characteristics of the studied women.  p.7 |
| *Data collection* |  |
| 17. Interview guide | Prior to data collection, the interview guide was reviewed by all study team members and piloted with 3 pregnant women who suspected to have fetus with anomaly. |
| 18. Repeat interviews | No |
| 19. Audio/visual recording | All interviews were audio recorded and transcribed verbatim so that the relevant themes could be reviewed by the research team. |
| 20. Field notes | The interviewer took notes during and after the interview. Interview notes were scanned and stored on a shared server, so team members could access, as needed. |
| 21. Duration | The interviews lasted for an average of 30 to 45 minutes. Data collection P.6 |
| 22. Data saturation | “3 consecutive interviews with no new themes”  P.6 |
| 23. Transcripts returned | A printed copy is returned to the participants to match the accuracy of the data with their experience. P.6 |
| **Domain 3: analysis and ﬁndings** |  |
| *Data analysis* |  |
| 24. Number of data coders | Six coders coded each interview. Each coded interview was reviewed by the FM.  . P.6 |
| 25. Description of the coding tree | N/A |
| 26. Derivation of themes | Themes were both hypothesized in advanced and derived from the data or conceptualized based on the data.  Results, p.6 |
| 27. Software | MAXQDA software (version 18) |
| 28. Participant checking | No |
| *Reporting* |  |
| 29. Quotations presented | Yes. Each quotation is identified with an individual code. Result.p.7 |
| 30. Data and ﬁndings consistent | Yes  Results, p.7-17 |
| 31. Clarity of major themes | Yes  Results, p.7-17 |
| 32. Clarity of minor themes | Yes  Results, p.7-17 |
